# Cardiogenic Shock: A Rare Complication of Influenza

**DOI:** 10.7759/cureus.2549

**Published:** 2018-04-29

**Authors:** Arsalan Talib Hashmi, Muhammad Sohail Yousuf, Husnain Waseem, Paurush Ambesh, Daniel Rodriguez, Aleksander Adzic

**Affiliations:** 1 Internal Medicine, Maimonides Medical Center, New York, USA; 2 Cardiology, Maimonides Medical Center, New York, USA

**Keywords:** influenza, cardiogenic shock, myopericarditis, congestive heart failure

## Abstract

We have presented a case of 41-year-old male who presented to the hospital with worsening shortness of breath, fatigue and flu-like symptoms. On admission to hospital, the patient was in severe cardiogenic shock secondary to acute perimyocarditis. He was admitted to the cardiac intensive care unit for close monitoring and aggressive hemodynamic support. Influenza B antigen was detected in nasopharyngeal aspirate and the patient was started on oseltamivir. The patient’s cardiac function improved significantly in few days and he was discharged home in stable condition with normal ejection fraction.

## Introduction

Perimyocarditis or myopericarditis refers to inflammation involving pericardial sac and myocardium. Acute pericarditis and myocarditis have similar causative agents and most cases in developed countries are caused by viruses. Most commonly involved viruses are coxsackieviruses, adenoviruses, and herpes viruses [1]. Influenza A virus is also a well-known cause of perimyocarditis; however, severe myocarditis and cardiogenic shock caused by influenza B are extremely rare. We describe a man with severe cardiogenic shock caused by influenza B-related myocarditis.

## Case presentation

A 41-year-old man with a past surgical history of uncomplicated cholecystectomy two years ago (and no other significant medical history) presented to emergency department with worsening fatigue, shortness of breath, and chest pain. He reported a one-week history of flu-like symptoms i.e. subjective fevers, cough, rhinorrhea, muscle aches, and two days history of pleuritic chest pain worsened by lying flat and improved by leaning forward. On day of presentation, he was feeling more fatigued and also had an episode of presyncope with chills and rigors. On arrival, physical examination revealed tachycardia to 106/minute, hypotension to 62/48 mmHg, and oral temperature of 97.9 °F.

On cardiac auscultation, no gallops or murmurs were appreciated. Lung auscultation revealed decreased air entry at right lung base and bibasilar crackles. No pathological findings were noted on abdominal exam.

**Figure 1:**
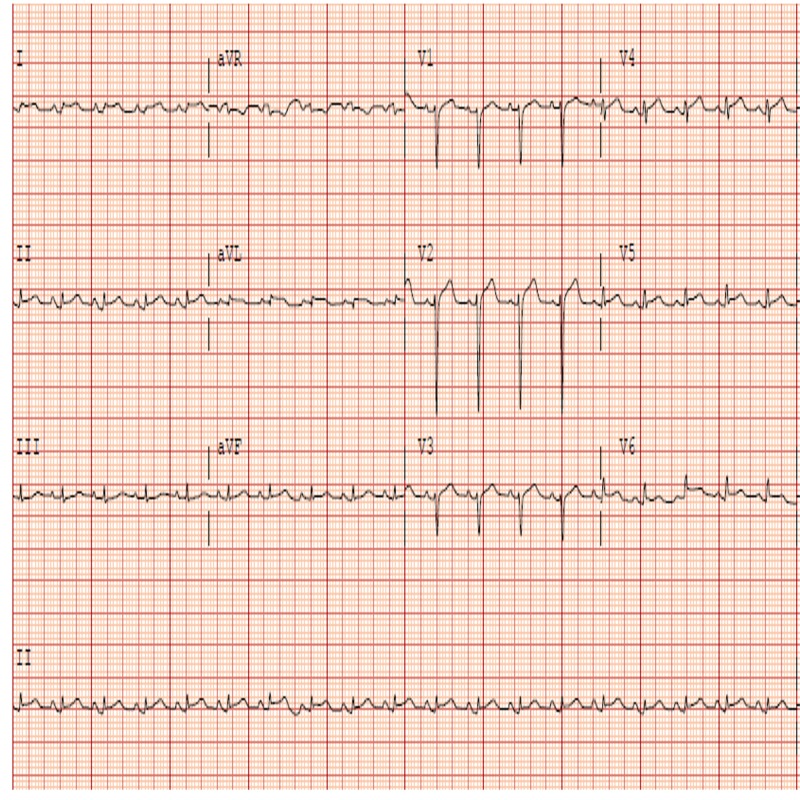
Electrocardiogram on admission showing diffuse ST elevations and PR depression

Electrocardiogram (ECG) showed sinus tachycardia and diffuse ST segment elevations and PR segment depressions except in lead aVR consistent with acute pericarditis (Figure 1).

**Figure 2:**
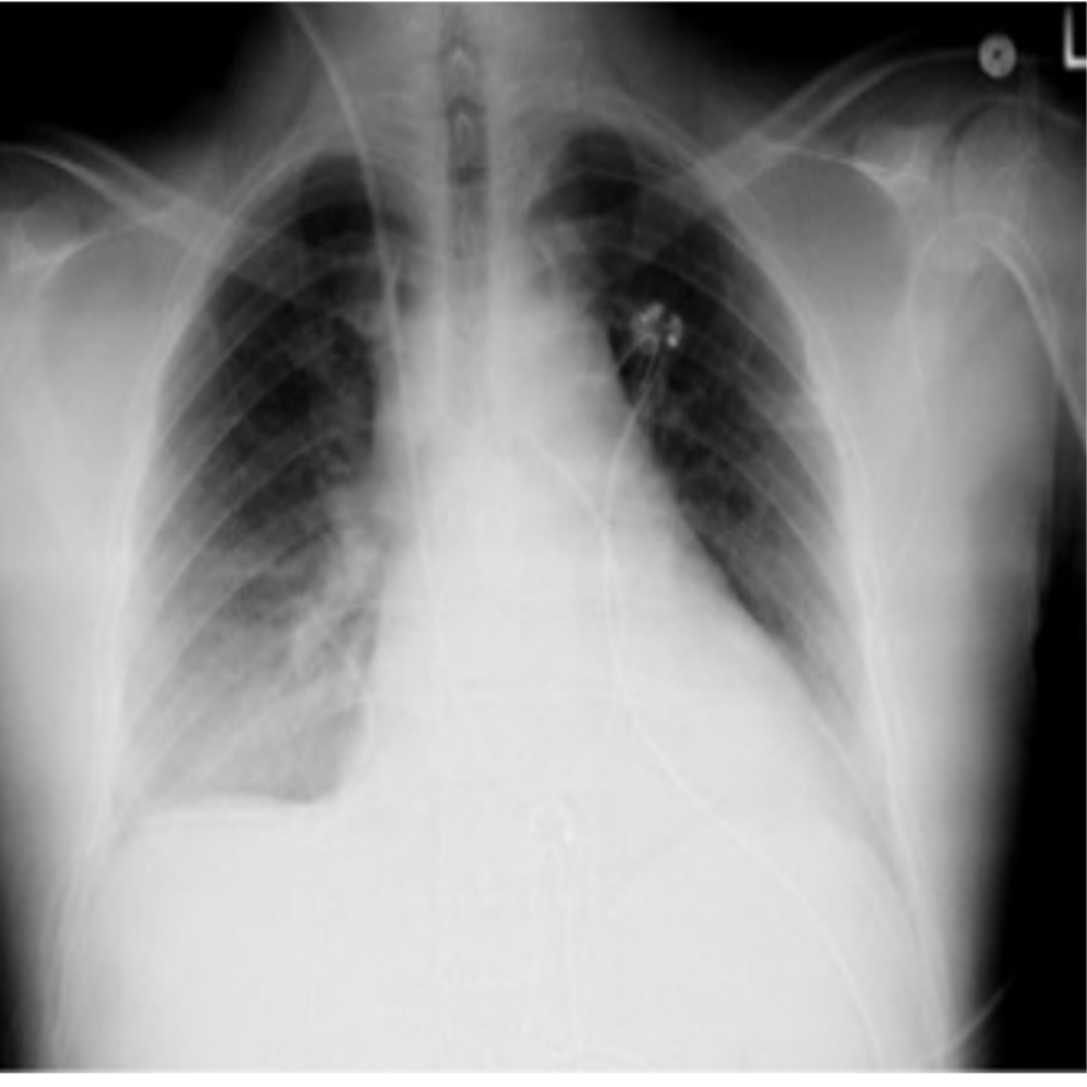
Chest X-ray showing pulmonary vascular congestion and right mid and lower lung opacity/effusion.

The patient was given 3 l of normal saline without significant improvement in hemodynamics. He was then started on vasopressors through the central line. Initial labs were significant for troponin I elevation to 2.39 ng/ml (ref 0.00-0.04), CK-MB 12.8 ng/ml (ref 0.6-6.3) CRP 2.637 mg/dl (ref 0.02-2.0), Ferritin 1473.9 ng/ml (ref 3.1-110.9). Chest X-ray showed pulmonary vascular congestion and right mid- and lower-lung opacity/effusion (Figure 2).

Bedside echocardiogram (ECHO) revealed severely reduced ejection fraction (EF) to 16%-20% and moderate pericardial effusion, which was later confirmed with the official echocardiogram as shown in Video 1.

**Video 1:** Echocardiogram on admission showing severely decreased left ventricular systolic function and moderate size pericardial effusion.

The patient was taken to cardiac intensive care unit for close hemodynamic monitoring. He was started on milrinone drip in addition to norepinephrine. Anti-inflammatory therapy with aspirin and colchicine were initiated. He was also started on Oseltamivir after rapid diagnostic test came back positive for Influenza B. The patient was able to be tapered off vasopressors and inotropes on day three. Repeat ECHO on day three of admission showed improved ejection fraction (EF) to 31 % and worsening pericardial effusion without tamponade effect. The hospital stay was complicated by paroxysmal atrial fibrillation and the patient was started on amiodarone for rhythm control. He was also started on heart failure medications i.e. lisinopril, metoprolol. Anticoagulation was not started due to low CHADS-Vasc score and risk of hemorrhagic conversion of pericardial effusion.

**Figure 3:**
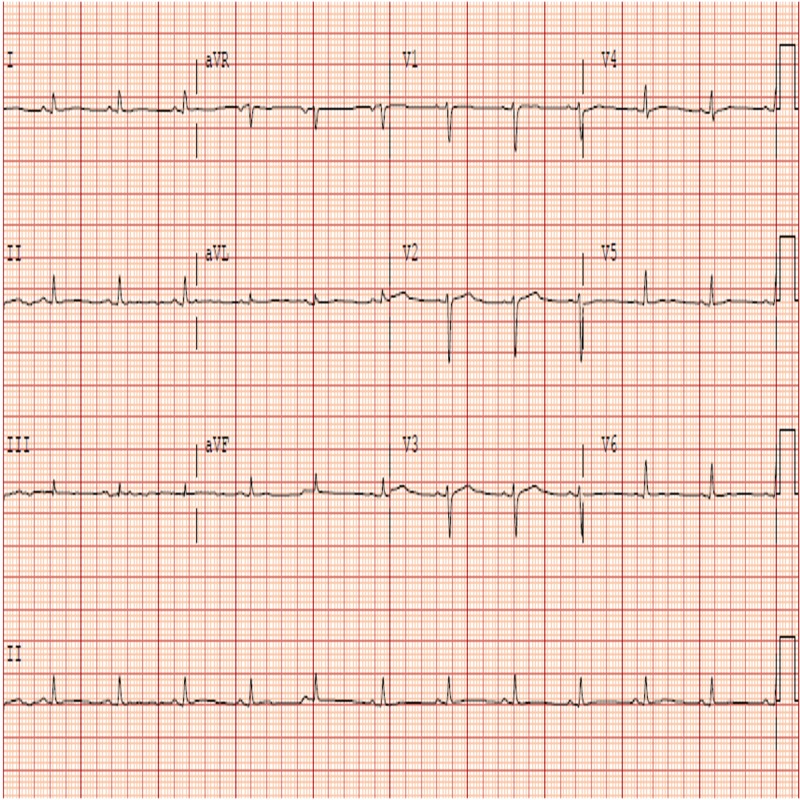
Electrocardiogram on day seven showing normalization of PR and ST segments

The patient remained in sinus rhythm afterward and was transferred from intensive care unit to telemetry floor. Follow-up ECG showed normalization of ST and PR segments (Figure 3).

Repeat Echocardiogram on day nine showed improved EF to 51% and resolution of pericardial effusion as shown in Video 2.

**Video 2:** Echocardiogram on discharge showing improved left ventricular systolic function and resolution of pericardial effusion.

 His symptoms resolved completely and he was discharged on day 10 in stable condition from the hospital to follow up with cardiology outpatient.

## Discussion

Acute myocarditis is a well-known complication of upper respiratory tract viral infection. The clinical expression varies from asymptomatic to fulminant myocarditis, which can result in severe hemodynamic compromise requiring high dose catecholamines and mechanical circulatory support. Pathogens frequently associated with myocarditis include coxsackievirus and adenovirus. Fulminant myocarditis resulting from influenza B viral infection is very rare but has been reported. Fifty-eight cases of myocarditis were reported during the famous 2009 pandemic of H1N1 (a strain of influenza A). Manhaz et al. described a case of 50-year-old woman with no history of heart disease who presented with profound cardiogenic shock with left ventricular ejection fraction of 10% with preceding symptoms of fever, chills, myalgias, and fatigue. An endotracheal aspirate was positive for influenza B. She had to be placed on extracorporeal membrane oxygenation but finally was able to go home on day fifteen with normal ejection fraction of 60% [2]. Frank et al. describe a case of a five-year-old girl who developed fulminant myocarditis due to acute infection with influenza virus type B. Cardiac arrest occurred suddenly, resuscitation efforts were unsuccessful, and the patient died of congestive heart failure 24 hours after the hospital admission [3].

Influenza B as compared to coxsackieviruses has low affinity for cardiac myocytes. The pathological effects of influenza myocarditis are more localized than seen in coxsackievirus myocarditis. After receptor-mediated binding and endocytosis of the virus in cardiomyocytes, there is infiltration of inflammatory cells in myocardium leading to cardiomyocyte necrosis and injury is continued by cytokine production and activation of cell-mediated immunity. Pan et al. investigated the molecular mechanism of myocarditis associated with influenza and revealed the importance of trypsin induction and increased production of matrix metalloproteinases and proinflammatory cytokines in the pathogenesis of acute myocarditis [4-7].

EKG is a sensitive and convenient tool for diagnosis of myopericarditis where ST elevation, T-wave inversion, and conduction block are observed. Continuous EKG monitoring also helps in the detection of fatal arrhythmias. ECHO findings include observation of transient wall thickness, reduced wall motion, reduced chamber size, and pericardial effusion. ECHO can also distinguish fulminant myocarditis from acute myocarditis. Felker et al. reported that patients with fulminant myocarditis had near normal diastolic dimensions with increased septal thickness, while those with acute myocarditis had increased diastolic dimensions with normal septal thickness [8].

Myocarditis is confirmed by findings of transient elevation of cardiac enzymes. Endomyocardial biopsy can be performed after ruling out coronary artery disease, although it is not essential for the clinical diagnosis of myocarditis. Takeuchi et al. reported that MRI might be more useful than invasive cardiac biopsy for diagnosing influenza myocarditis [9]. Magnetic resonance imaging (MRI) identifies intramyocardial inflammation of influenza myocarditis depicted as nodular delayed enhancement in a diffuse, predominantly infero-lateral sub-epicardial location in non-vascular territories.

Traditionally, viral infection is diagnosed if the viral antibody titer is at least four times higher in an acute phase serum sample than that in a sample obtained in the remission phase (obtained two weeks apart); newer diagnostic techniques of rapid viral diagnosis from nasal swabs are now readily available.

Treatment of influenza myocarditis should be directed to eliminate the cause, improve cardiac function and hemodynamic compromise. It is recommended to treat all the patients of influenza B myocarditis with neuraminidase inhibitors like oseltamivir and zanamivir. The low-case fatality rate in Japanese influenza pandemic can be a result of early aggressive treatment with these antiviral agents [10].

The first therapy for acute myocarditis patients with fulminant heart failure is supportive interventions with pharmacological/ mechanical means. Pharmacological support by vasopressors and inotropes should be used while the patient is being taken for interventions to provide mechanical support. Intra-aortic balloon pump (IABP), extracorporeal membrane oxygenation (ECMO) and left ventricular assist device (LVAD) are the different assist devices that can be used as a bridge to transplantation in potentially fatal cases where EF does not improve. Although an acute drop in EF is an indication for left heart catheterization to evaluate and treat coronary artery disease; however, young patients without significant risk factors for ischemic heart disease and clinical presentation consistent with myopericarditis do not require coronary angiogram. Patients which show an improvement in EF but to near normal levels should be placed on beta-blockers, angiotensin converting enzyme inhibitors and diuretics (to decrease pre and afterload to assist the heart function) with periodic monitoring of their EF every three to six months.

## Conclusions

Influenza B myocarditis is generally considered to be less severe; however, fulminant myocarditis and cardiogenic shock are possible complications. We recommend early diagnosis by improved methods of rapid diagnostic test, polymerase chain reaction (PCR) assay, EKG, ECHO, and Cardiac MRI. Aggressive cardiac support and early antiviral therapy are the key to better outcomes.

## References

[1] Imazio M, Trinchero R (2008). Myopericarditis: etiology, management, and prognosis. Int J Cardiol.

[2] Taremi M, Amoroso A, Nace HL, Gilliam BL (2013). Influenza B-induced refractory cardiogenic shock: a case report. BMC Infect Dis.

[3] Frank H, Wittekind C, Liebert UG (2010). Lethal influenza B myocarditis in a child and review of the literature for pediatric age groups. Infection.

[4] Pan HY, Yamada H, Chida J (2011). Up-regulation of ectopic trypsins in the myocardium by influenza A virus infection triggers acute myocarditis. Cardiovasc Res.

[5] Wang S, Le TQ, Kurihara N (2010). Influenza virus-cytokine-protease cycle in the pathogenesis of vascular hyperpermeability in severe influenza. The Journal of infectious diseases.

[6] Wang S, Quang Le T, Chida J (2010). Mechanisms of matrix metalloproteinase-9 upregulation and tissue destruction in various organs in influenza A virus infection. J Med Invest.

[7] Teijaro JR, Walsh KB, Cahalan S (2011). Endothelial cells are central orchestrators of cytokine amplification during influenza virus infection. Cell.

[8] Felker GM, Boehmer JP, Hruban RH (2000). Echocardiographic findings in fulminant and acute myocarditis. J Am Coll Cardiol.

[9] Takeuchi I, Imaki R, Inomata T, Soma K, Izumi T (2010). MRI is useful for diagnosis of H1N1 fulminant myocarditis. Circ J.

[10] Sugaya N (2011). Widespread use of neuraminidase inhibitors in Japan. J Infect Chemother.

